# *learnPopGen*: An R package for population genetic simulation and numerical analysis

**DOI:** 10.1002/ece3.5412

**Published:** 2019-07-01

**Authors:** Liam J. Revell

**Affiliations:** ^1^ Department of Biology University of Massachusetts Boston Boston Massachusetts USA; ^2^ Departamento de Ecología, Facultad de Ciencias Universidad Cátolica de la Santísima Concepción Concepción Chile

**Keywords:** evolutionary biology, simulation, teaching

## Abstract

Here, I briefly present a new R package called *learnPopGen* that has been designed primarily for the purposes of teaching evolutionary biology, population genetics, and evolutionary theory. Functions of the package can be used to conduct simulations and numerical analyses of a wide range of evolutionary phenomena that would typically be covered in advanced undergraduate through graduate‐level curricula in population genetics or evolution. For instance, *learnPopGen* functions can be used to visualize gene frequency changes through time under multiple deterministic and stochastic processes, to compute and animate the changes in phenotypic trait values or distributions under natural selection, to numerically analyze and graph the outcome of simple game theory models, and to plot coalescence within a population experiencing genetic drift, along with a number of other things. Functions have been designed to be maximally didactic and frequently employ compelling animated visualizations. Furthermore, it is straightforward to export plots and animations from R in the form of flat or animated graphics, or as videos. For maximum flexibility, students working with the package can run functions directly in R; however, instructors may choose to guide students less adept in the R environment to one of various web interfaces that I have built for a number of the functions of the package and that are already available online.

## 1 INTRODUCTION

In this short article, I present a new R package called *learnPopGen* that I have developed with the expressed purpose of teaching (and/or learning about) population genetics, quantitative genetics, and evolutionary theory. R (R Core Team, 2019) is a scientific computing environment that is commonly taught to biology majors at institutions of higher education worldwide. Much of the diverse functionality of R arises from its contributed packages, built by individual scientists and software developers outside of the core R team. The *learnPopGen* package arises from a series of functions that I developed (and continue to develop) in fits and starts over the course of my nine or so years as a university‐instructor and that I have used both in undergraduate and graduate‐level pedagogy. To date, I have personally employed functions from this package in teaching courses or seminars in *Evolution*, *Evolutionary Theory*, and *Animal Behavior*; however, I anticipate that the functionality of the package could be of significant use in undergraduate or graduate courses across a variety of other disciplines, but particularly in which themes from population or quantitative genetics are covered. Although there has been no prior publication describing this package, the source code has been available online via my *GitHub* page for several years (previously under the alternative moniker, *PopGen*). Consequently, a number of colleagues have already reported using functions of the package in their own teaching.

In this short article, I will briefly describe the history of development of *learnPopGen* as well as the range of functions that presently exist in the package. Meanwhile, I will mention a few words about what I envision as being typical use of *learnPopGen* in instruction. Finally, I will describe the web interfaces that I have developed for a significant number of the package functions.

## 2 DESCRIPTION AND IMPLEMENTATION

### 2.1 Description of the package

The *learnPopGen* package is at its core a library of different R functions designed to be used in teaching and learning key concepts in evolutionary biology, evolutionary theory, and population genetics. Though I had never described the package in a formal publication until now, the functionality of *learnPopGen* has been years in the making. I first developed the initial series of functions of this package as part of an informal graduate seminar that I organized many years ago to review the excellent book *Evolutionary Theory: Mathematical and Conceptual Foundations* (Rice, 2004). The functions that I developed at this time conduct both numerical analyses of relatively simple mathematical models along with some stochastic simulations. These functions were originally implemented for a different scientific computing environment; however, I subsequently translated all of them to run in R and they are now incorporated into *learnPopGen*. They range from a simple genotypic selection model, through frequency‐dependent selection, through a model of natural selection and mutation, through a function for genetic drift, among others. All functions exported to the namespace at the time of writing (and thus directly available for package users) are listed and annotated in Table 1.

**Table 1:** Annotated list of all the functions of the *learnPopGen* package at the time of writing

Function name	Description	Uses animation?	Web interface?
clt	Illustrates the concept of the central limit theorem by plotting the sum or mean of random variables with different underlying distributions	No	Yes
coalescent.plot	Creates a simulation of allele coalescence within a population and plots a genealogy of alleles through time	Yes	Yes
drift.selection	Simulates simultaneous genetic drift and natural selection at a biallelic locus and graphs the result	Yes	Yes
founder.event	Simulates a founder event or population bottleneck and graphs the result	No	Yes
freqdep	Numerically analyzes a frequency‐dependent selection model and produces one of various graphs, including changes in allele frequencies through time	Yes	Yes
genetic.drift	Simulates genetic drift alone and plots the change in allele frequency through time	Yes	Yes
hardy.weinberg	Computes and plots the Hardy–Weinberg frequencies for a multiallelic locus	No	No
hawk.dove	Conducts numerical analysis of a hawk‐dove game‐theoretic model and graphs the resultant change in each strategy through time	No	Yes
msd	Simulates simultaneous migration, selection, and genetic drift within and between two populations and graphs the changes in allele frequency through time in each population	No	Yes
multilocus.hw	Computes and plots Hardy–Weinberg frequencies for multiple biallelic loci	No	Yes
mutation.selection	Conducts numerical analysis of gene frequencies through time under mutation–selection balance and graphs the result	No	No
phenotype.freq	Computes and plots the phenotypic trait distribution for a polygenic trait under certain simplifying assumptions	No	Yes
phenotype.selection	Computes and animates the change in phenotypic trait distribution for a polygenic trait under directional natural selection	Yes	No
rcd	Simulates and graphs reproductive character displacement in an ecological community*	No	No
selection	Performs numerical analysis of a frequency‐independent biallelic selection model and produces one of various graphs, including the change in allele frequency through time	Yes	Yes
sexratio	Numerically analyzes a hypothetical model of frequency‐dependent selection on a sex‐determining genetic locus and plots the result	Yes	No

* rcd( ) was developed as part of another study, but for convenience and to avoid the unnecessary proliferation of R libraries on CRAN has been packaged with the *learnPopGen* R library.

**Figure 1:**
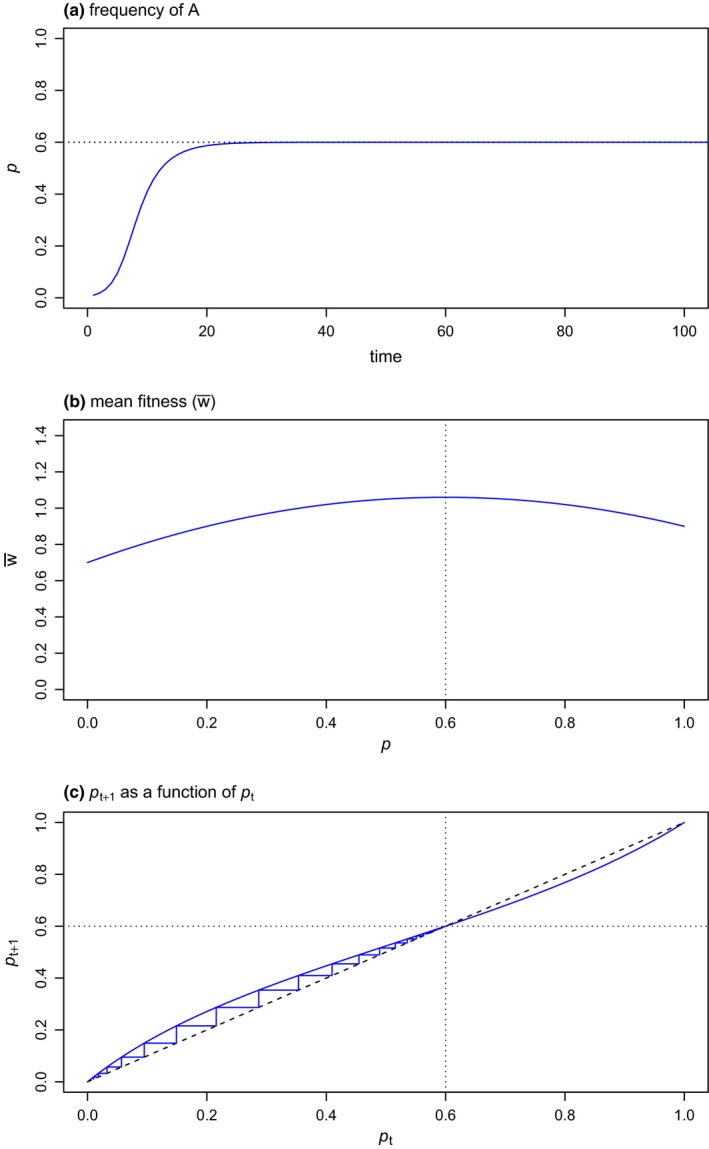
Numerical analysis of natural selection on a biallelic locus under a scenario of overdominance for fitness (i.e., in which$w\left(\mathrm{Aa}\right)>w\left(\mathrm{AA}\right)\geq w\left(\mathrm{aa}\right)$). The three panels of the figure show three different plots that were produced using the function *selection* of the *learnPopGen* package. (a) The relative frequency of the *A* allele through time. (b) A fitness landscape showing mean fitness ($\bar{w}$) as a function of the allele frequency, *p*. (c) A “cobweb plot” showing *p_t_*_+1_ as a function of *p_t_*. Equilibrium values of *p* under the model are given by the plotted vertical or horizontal dotted lines

To give one example, the function *selection* conducts numerical analysis of a simple genotypic selection model. The user must first specify relative fitness values for three genotypes: *AA*, *Aa*, and *aa*, an initial frequency of the *A* allele in the population (*p*_0_), and a number of generations over which to analyze the model. The user can then choose to visualize a variety of different results: the frequency of the *A* allele (denoted *p*) as a function of time; the frequency of *a* (denoted *q*); mean fitness, $\bar{w}$, through time; $\bar{w}$ as a function of *p* (i.e., the fitness landscape); the change in *p* between generations (∆*p*) as a function of *p*; and, finally, a so‐called “cobweb plot” showing *p_t_*_+1_ as a function of *p_t_* as the frequency of *A* evolves toward fixation or equilibrium (Rice, 2004). Figure 1 gives three of these plots for a rather extreme scenario of overdominance for fitness in which *Aa* individuals have higher fitness than either homozygous genotype. Though shown statically in Figure 1, this function and others like it in the package can also be animated such that the frequency of *A* grows or declines as evolution proceeds, or such that the steps in the cobweb plot are added sequentially with time (Table 1). For use in lectures, static plots can be exported from R as high‐quality vector or raster images, or as animated GIFs with the help of the open‐source software *ImageMagick* (http://www.imagemagick.org/; Cristy, 1990), which can easily be called from within R.

Much later, while teaching *Animal Behavior*, I developed a function to illustrate a simple example of the *hawk‐dove* game (Maynard Smith & Price, 1973). For those unfamiliar with this game, the general idea is not that “hawk” and “dove” are different animal species—but, rather, that they are two competing behavioral strategies that co‐occur in a population: roughly akin to “fight” and “share,” respectively. According to the most common parameterization of this model, average fitness is highest when all members of the population adopt the dove strategy, but this scenario is evolutionarily unstable because a population consisting entirely of doves is highly invasible by the alternative, hawk strategy. By contrast, a high population frequency of the hawk strategy results in lower mean fitness, but is evolutionarily stable because such a population cannot be invaded by doves. Though simplistic, the model nicely illustrates the concept of “evolutionarily stable strategies” and the idea that evolution by natural selection does not always favor the phenotype that maximizes population mean fitness.

In this game, the user must specify an initial frequency of “hawks,” as well as a pay‐off matrix for interactions between hawks and doves. The function then evolves the population, assuming that all hawks and doves interact in exactly the proportions that they are represented in the population and that their relative fitnesses are determined by the interaction outcomes specified in the pay‐off matrix.

**Figure 2:**
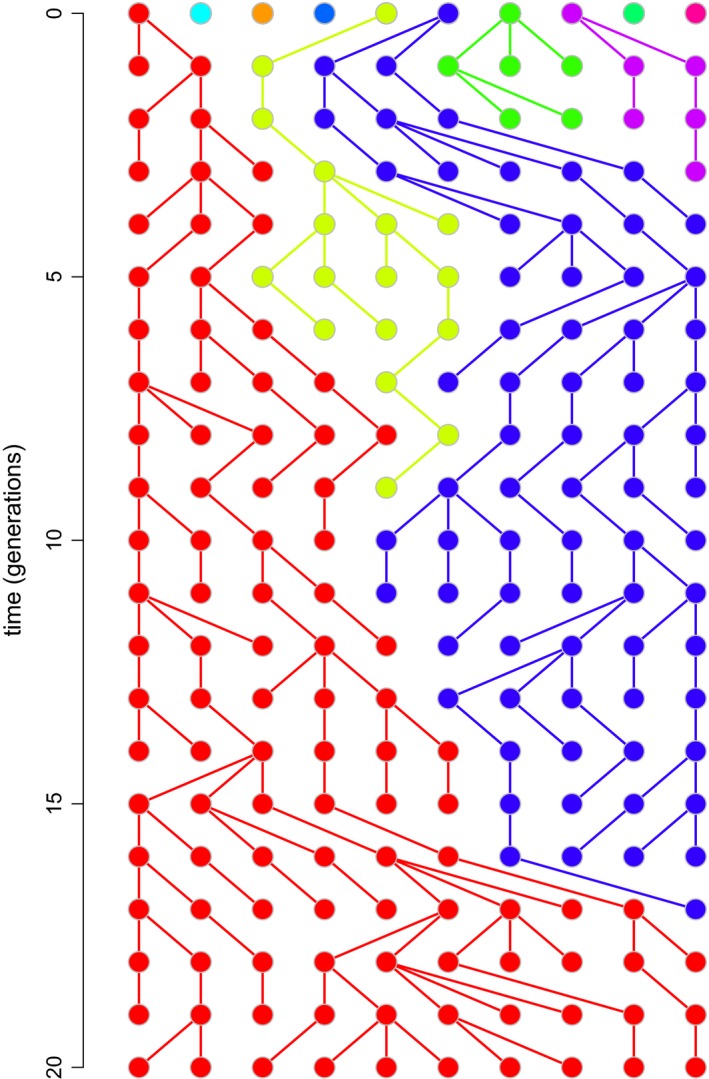
A genealogy of alleles in a Wright–Fisher population evolving by genetic drift created using the function *coalescent.plot* of *learnPopGen*. In an interactive R session, this graphic would be animated and can be generated for an arbitrary number of generations and gene copies

Finally, and most recently, while teaching *Evolution* at the undergraduate level, I developed an additional series of functions to illustrate a number of other key concepts in population and quantitative genetics. For instance, I developed a function called *phenotypic.selection* that demonstrates via animation how directional selection can take the phenotypic distribution of a genetically based polygenic trait far outside of its original range, even absent the introduction of new genetic variation via mutation. Another function, *drift.selection*, can be used to simulate the simultaneous effects of selection and drift at a biallelic locus. Another function still, *coalescent.plot*, illustrates the concept of genetic coalescence (Kingman, 1982) in a Wright–Fisher population of alleles experiencing genetic drift. An example of a simulation produced via *coalescent.plot* is given in Figure 2.

**Figure 3:**
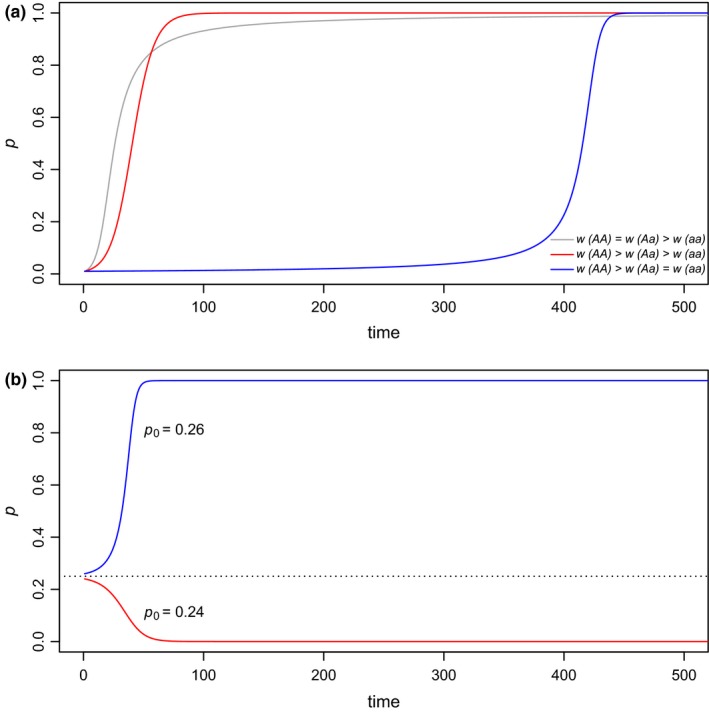
The frequency of allele *A* (*p*) as a function of time under several different scenarios computed and graphed using the function *selection* in the *learnPopGen* package. (a) Dominance for fitness (i.e., $w\left(\mathrm{AA}\right)=w\left(\mathrm{Aa}\right)>w\left(\mathrm{aa}\right)$), codominance for fitness (i.e., $w\left(\mathrm{AA}\right)>w\left(\mathrm{Aa}\right)>w\left(\mathrm{aa}\right)$), and dominance of *a* for fitness (i.e., to say, a recessive fitness advantage of the *A* allele, i.e., $w\left(\mathrm{AA}\right)>w\left(\mathrm{Aa}\right)=w\left(\mathrm{aa}\right)$). (b) Underdominance for fitness ($w\left(\mathrm{AA}\right)>w\left(\mathrm{Aa}\right)$ and $w\left(\mathrm{aa}\right)>w\left(\mathrm{Aa}\right)$). In (b), the equilibrium (indicated by the dotted line) is unstable, and whether allele *A* or *a* is fixed depends on their initial frequencies

I anticipate that many readers of this article are more creative than I and are thus likely to think of numerous more interesting ways to make use of the functions of *learnPopGen*. My general vision for this type of pedagogical tool, however, is to transform cold equations into dynamic, interactive models. To take a simple case, most readers of this article probably vaguely recall studying the simple biallelic selection model expressions in their introductory evolution or population genetics classwork. These formulae are relatively dry and lifeless, yet they nonetheless lead to several very important and meaningful predictions about how evolution should proceed depending on the relative fitnesses of the *AA*, *Aa*, and *aa* genotypes. For instance, assuming selection is frequency independent, coexistence of both alleles at equilibrium is expected *only* under conditions of overdominance for fitness (e.g., when $w\left(\mathrm{Aa}\right)>w\left(\mathrm{AA}\right)\geq w\left(\mathrm{aa}\right)$; Figure 1a). Complete dominance for fitness (i.e., $w\left(\mathrm{AA}\right)=w\left(\mathrm{Aa}\right)>w\left(\mathrm{aa}\right)$), coupled with a low starting frequency of the *A* allele, will result in an initial rapid increase in *A*, but progress toward fixation will slow through time (compared to a codominant model in which $w\left(\mathrm{AA}\right)>w\left(\mathrm{Aa}\right)>w\left(\mathrm{aa}\right)$) as fewer and fewer *a* alleles are found in homozygous individuals of low fitness (Figure 3a). By contrast, if *a* has a dominant negative effect on fitness (i.e., $w\left(\mathrm{aa}\right)=w\left(\mathrm{Aa}\right)<w\left(\mathrm{AA}\right)$, i.e., the positive fitness effect of *A* is *recessive*), and *A* is initially rare, than progress toward fixation of *A* will be at first retarded when almost all *A* alleles are found in heterozygotes, but then should accelerate rapidly as *A* reaches higher and higher frequency the population. This is because all *a* alleles result in low fitness (regardless of the genotype in which they are found) and thus are relatively easy to purge via natural selection (Figure 3a). Finally, underdominance for fitness (i.e., $w\left(\mathrm{aa}\right)>w\left(\mathrm{Aa}\right)$ and $w\left(\mathrm{AA}\right)>w\left(\mathrm{Aa}\right)$) produces an *unstable* equilibrium in which either allele could evolve to fixation depending on its initial frequency (Figure 3b).

Even this relatively simple function can be used to confront common learning difficulties that students may have when encountering allelic selection models. For instance, Soderberg and Price (2003) point out that students often tend to associate the term “dominance” with superiority or greater vigor, even though dominance (in genetic parlance) merely refers to the phenotype of the heterozygote. They will thus assume that selection will result in more rapid fixation of a dominant allele, when (in fact) this is only true if the allele is at low frequency in the population. As the dominant allele rises in frequency in the population (or if its initial frequency is set to be >0.5), then students will quickly find that selection is actually less efficient at fixing a positively selected dominant allele than a codominant or recessive allele (Soderberg & Price, 2003). The same concept is presented statically (although, in my opinion, very clearly) in a commonly used textbook (Futuyma & Kirkpatrick, 2017). I believe that a teaching module in which students are permitted to explore the parameters of the model and their effects in an interactive setting could nonetheless lead to a more profound understanding and more effective internalization of fundamental concepts for some learners.

In my opinion, in a maximally didactic class exercise students would explore these alternative models to understand how evolutionary dynamics are expected to proceed in every case. Then, the instructor could challenge them to explain each of the different outcomes they uncover. Alternatively, in a scenario in which only the instructor runs R or interacts with the web interface of the function, students could be asked to predict and vote a priori on how they expect evolution to proceed in each of the aforementioned scenarios, as well as others, and then their predictions could be immediately validated or refuted via numerical analysis of each model by the instructor.

### 2.2 Description of the web interfaces

In addition to the R library itself, I have also built a number of web interfaces to various functions of the *learnPopGen* package. These web interfaces were developed using the *shiny* (Chang, Cheng, Allaire, Xie, & McPherson, 2017) web application framework for R. Using *shiny* web interfaces in lieu of R to run the functions of this package (or at least those for which web interfaces have been developed, see Table 1) means that students (and instructors) need no prior R experience. Only a basic familiarity with standard web browser elements (action buttons, sliders, text boxes, and so on) is required. The web interface option may also be useful for a classroom setting in which each student does not have access to a computer, as the web applications run easily from a typical smartphone.

**Figure 4:**
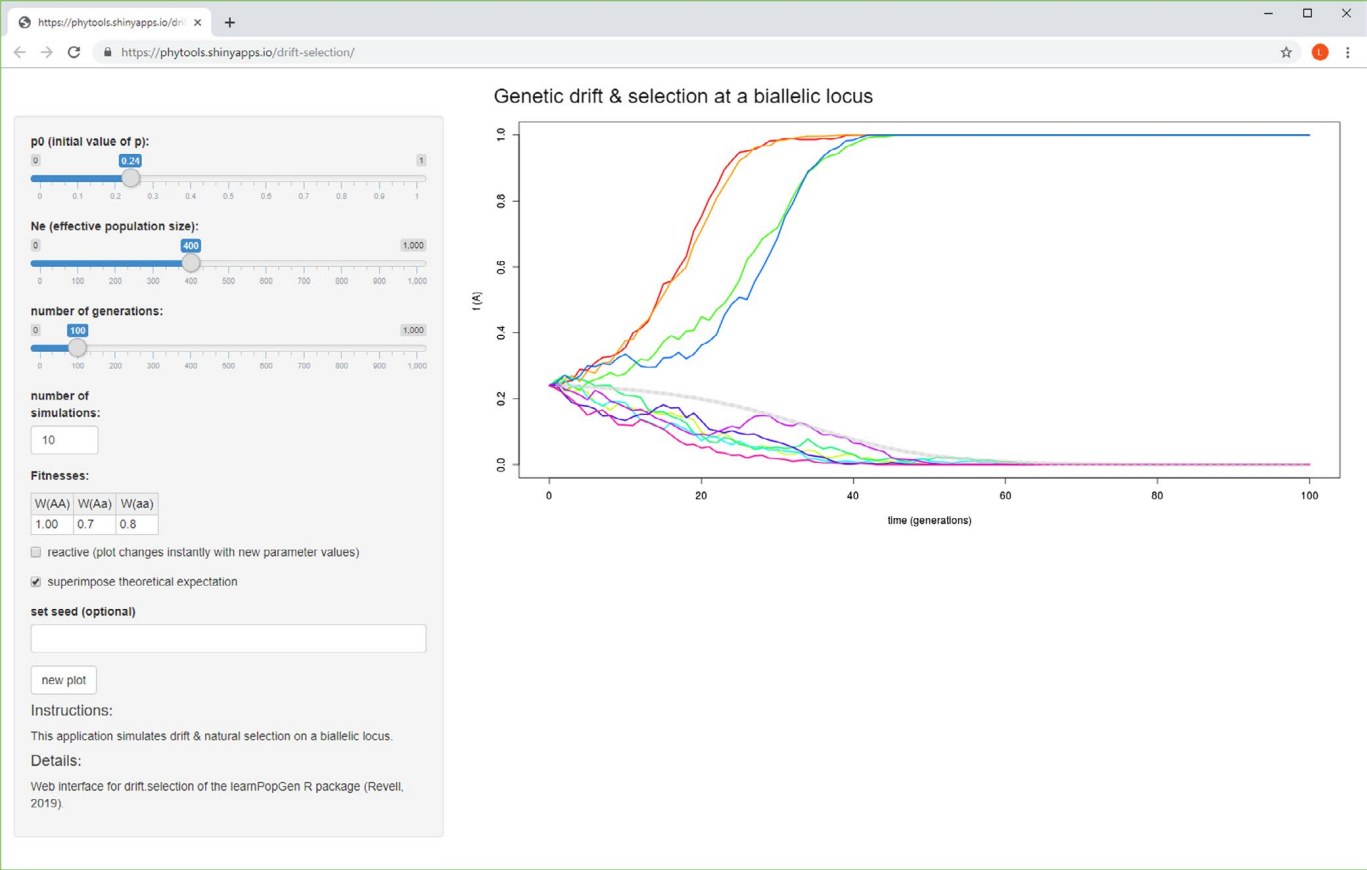
A screenshot of the *shiny* (Chang et al., 2017) web interface for *drift.selection* in the *learnPopGen* package

Users preferring to run the functions of *learnPopGen* via their web interfaces have two options. The simpler of these is to access the functions via the web page, as previously mentioned: http://www.phytools.org/PopGen. All of the interfaces on this page can be controlled via a web browser, but are executed on a remote server with R and *learnPopGen* installed. (Currently, this server is http://shinyapps.io via a subscription service paid by me.) Alternatively, for users with R, *shiny*, and *learnPopGen* installed locally, it is also possible to control the same functions via a web browser (but not over the Internet) by simply downloading the source code of the web applications from their GitHub page (http://github.com/liamrevell/PopGen.apps), and then executing the application source within *Rstudio* (Rstudio Team, 2015) or by using the *shiny* function *runApp*. Figure 4 shows an example web interface for the function *selection.drift*. A notable disadvantage of using the web interfaces (i.e., instead of running the functions of the package in an interactive R session) is that, at least at the time of writing, plotted graphics in the web interfaces cannot be animated. They are, however, frequently “reactive” (i.e., to say, when the arguments of the function change, the analysis is automatically recomputed and replotted).

### 2.3 Notes on implementation and installation

All the functions of *learnPopGen* have been implemented for the R statistical computing environment (R Core Team, 2019), and all simulations and analyses of this article were conducted in R. *learnPopGen* in turn depends on various packages of core R (*grDevices*, *graphics*, *methods*, *stats*) as well as on the additional packages *gtools* (Warnes, Bolker, & Lumley, 2018) and *phytools* (Revell, 2012).

Development of the aforementioned web interfaces of *learnPopGen* was undertaken using *shiny* (Chang et al., 2017) within Rstudio (Rstudio Team, 2015). *shiny* in turn depends on a number of other R packages (Chang, 2017; Cheng, 2018a, 2018b; Cheng, Corrada Bravo, Ooms, & Chang, 2018; Csárdi, 2017; Dahl, 2016; Eddelbuettel et al., 2018; Henry & Wickham, 2018; Ooms, 2014; Rstudio & Inc., 2017; Ushey, 2017; Xie, 2016). Certain of the *learnPopGen* web interfaces also use the R package *rhandsontable* (Owen, 2018), which too imports functionality from several other packages (Bache & Wickham, 2014; Ooms, 2014; Vaidyanathan, Xie, Allaire, Cheng, & Russell, 2018).

*learnPopGen* can be installed directly from its GitHub page (https://github.com/liamrevell/learnPopGen) using the package *devtools* (Wickham, Hester, & Chang, 2018) or from the Comprehensive R Archive Network, CRAN (https://CRAN.R-project.org/package=learnPopGen).

## 3 CONCLUSION

Herein, I present a new R package, *learnPopGen*, for teaching and learning about population genetics, evolutionary biology, quantitative genetics, and related disciplines. Many functions of the package employ compelling graphics and animations. Students can use the package within an interactive R session, or instructors can export plots and animations as flat graphics or animations for use in lecture. Finally, for cases in which the classroom setting or classroom time does not permit the use of R, I have also built a number of web interfaces for functions of the package which use R on a remote server but can be run from any Internet‐connected browser or smartphone.

## CONFLICT OF INTEREST

None declared.

## AUTHOR CONTRIBUTIONS

LJR conceived the project, undertook all aspects of its implementation, and wrote the manuscript.

## Data Availability

Software described in this article is available on GitHub (https://github.com/liamrevell/learnPopGen and https://github.com/liamrevell/PopGen.apps) as well as via the Comprehensive R Archive Network, CRAN (https://CRAN.R-project.org/package=learnPopGen).
